# Supplementary material for: Therapeutic potential of vitamin D against bisphenol A-induced spleen injury in Swiss albino mice
Source: PLoS One. 2023 Mar 9;18(3):e0280719. doi: 10.1371/journal.pone.0280719 (PMC9997876; doi:10.1371/journal.pone.0280719)
Supplement: S1 File — (PDF) [file pone.0280719.s001.pdf]

Author First, Quality First
www.enago.com

localize to globalize
www.ulatus.com

we are all ears
www.voxtab.com

CERTIFICATE OF EDITING

This is to certify that the paper titled Therapeutic potential of vitamin D against bisphenol A-induced spleen injury commissioned to us by Aziz Alsuqih has been edited for English language, grammar, punctuation, and spelling by Enago, the editing brand of Crimson Interactive Inc. under Copyediting B2C.

ISO 17100:2015
Translation Service Providers

ISO 27001:2013
Information Security Management System

ISO 9001:2015
Quality Management System

Issued by:
Enago, Crimson Interactive Inc.
160, Greentree Dr, Ste 101 street,
Dover City, Kent, Delaware, 19904
Phone: +1-302-498-8358

Disclaimer : The intent of the author's message has been preserved during the editing process. The author is free to accept or reject our changes in the document after reviewing our edits. This certificate has been awarded at the time of sharing the final edited version (full file or sections of the file) with the author. Enago does not bear any responsibility for any alterations done by the author to the edited document post .
