# Supplementary material for: Therapeutic potential of vitamin D against bisphenol A-induced spleen injury in Swiss albino mice
Source: PLoS One. 2023 Mar 9;18(3):e0280719. doi: 10.1371/journal.pone.0280719 (PMC9997876; doi:10.1371/journal.pone.0280719)

Figure 7

Histopathological analysis in hematoxylin and eosin (H&E) stained spleen of the BPA-treated mice and BPA+VitD-treated mice (40×).

BPA, male (10X)

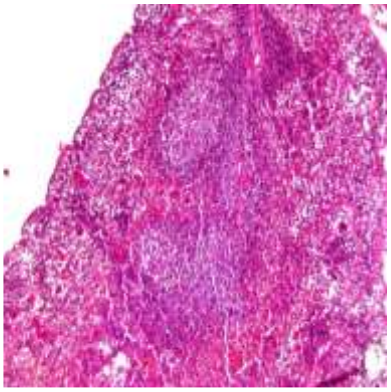

BPA, male (40X)

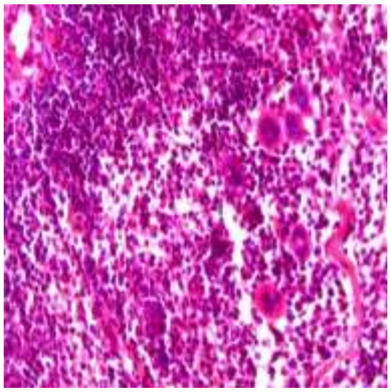

BPA, female (10X)

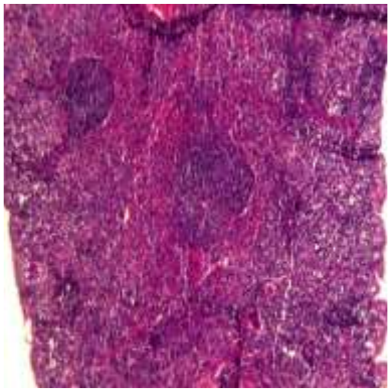

BPA, female (40X)

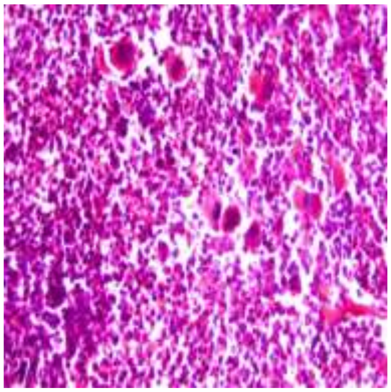

BPA+VitD, male (10X)

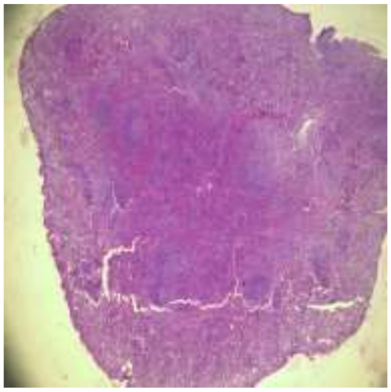

BPA+VitD, male (40X)

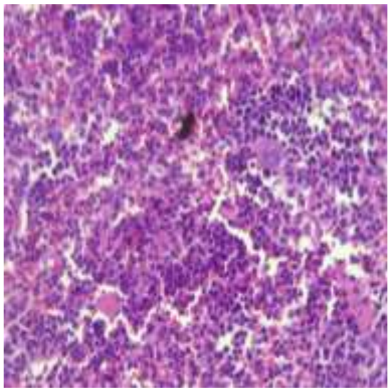

BPA+VitD, female (10X)

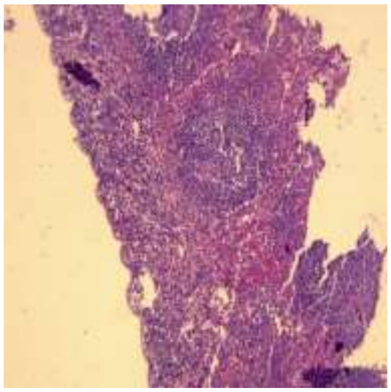

BPA+VitD, female (40X)

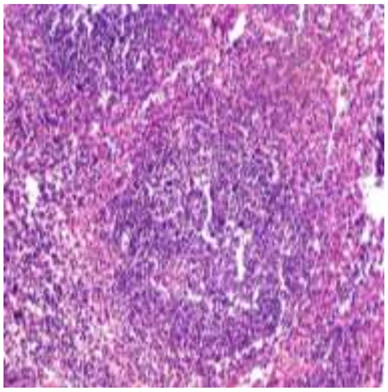

Supplement: S1 Raw images — (ZIP) [file pone.0280719.s002.zip › Figure 7, all images.pdf]
