# Supplementary figures and images for: Therapeutic potential of vitamin D against bisphenol A-induced spleen injury in Swiss albino mice
Source: PLoS One. 2023 Mar 9;18(3):e0280719. doi: 10.1371/journal.pone.0280719 (PMC9997876; doi:10.1371/journal.pone.0280719)

# Figure 8

VitD reduces the genomic DNA integrity in BPA-treated mice.

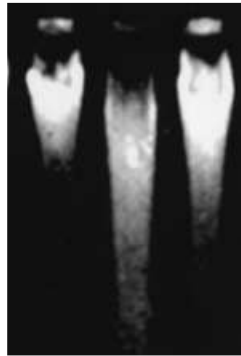

Supplement: S1 Raw images — (ZIP) [file pone.0280719.s002.zip › Figure 8, all images.pdf]
