# Supplementary material for: Therapeutic potential of vitamin D against bisphenol A-induced spleen injury in Swiss albino mice
Source: PLoS One. 2023 Mar 9;18(3):e0280719. doi: 10.1371/journal.pone.0280719 (PMC9997876; doi:10.1371/journal.pone.0280719)
Supplement: Attachment — Submitted filename: reviewer.docx [file pone.0280719.s003.docx]

The authors of the manuscript entitled “ **Therapeutic potential of vitamin D against bisphenol A induced spleen injury”** is well written and easy to understand. The manuscript could be considered for publication provided the authors can address major issues listed below.

1. In introduction the author mentioned about the negative effects of vitamin D, briefly explain the negative effects.
2. In material and methods author didn’t mention the detail information about the diet given to mice.
3. In statical analysis the author performed two way ANOVA analysis, a suggestion of performing one way ANOVA comparison within the groups
4. Why the author choose 50ug of BPA dose.
5. Mostly the BPA and Vitamin doses are administered orally, why the author choose to inject intraperitoneally.
6. The discussion is quiet long, it’s better to concise it.
