# Supplementary material for: Therapeutic potential of vitamin D against bisphenol A-induced spleen injury in Swiss albino mice
Source: PLoS One. 2023 Mar 9;18(3):e0280719. doi: 10.1371/journal.pone.0280719 (PMC9997876; doi:10.1371/journal.pone.0280719)
Supplement: Attachment — Submitted filename: Response to Reviewers 6.9.2022.docx [file pone.0280719.s004.docx]

PONE-D-22-20374

**Therapeutic potential of vitamin D against bisphenol A-induced spleen injury** **in Swiss albino mice**

Thanks for reviewers for their good efforts to examine our MS, and the following responses have been made regarding their comments:

**Journal Requirements:**

**When submitting your revision, we need you to address these additional requirements.**

1. **Please ensure that your manuscript meets PLOS ONE's style requirements, including those for file naming. The PLOS ONE style templates can be found at
   https://journals.plos.org/plosone/s/file?id=wjVg/PLOSOne_formatting_sample_main_body.pdf and** 
   [**https://journals.plos.org/plosone/s/file?id=ba62/PLOSOne_formatting_sample_title_authors_affiliations.pdf**](https://journals.plos.org/plosone/s/file?id=ba62/PLOSOne_formatting_sample_title_authors_affiliations.pdf)**.**

**Authoress’s Response**

The manuscript has been adjusted to meet PLOS ONE's style requirements

2. **comply with PLOS ONE submissions requirements, in your Methods section, please provide additional information regarding the experiments involving animals and ensure you have included details on (1) methods of sacrifice, (2) methods of anesthesia and/or analgesia, and (3) efforts to alleviate suffering.**

**Authoress’s Response**

- methods of sacrifice → The animals were sacrificed under anesthesia, and the
- methods of anesthesia and/or analgesia → with 1% ketamine for dissection
- efforts to alleviate suffering → reduce no of animal used

3. **We note that you have indicated that data from this study are available upon request. PLOS only allows data to be available upon request if there are legal or ethical restrictions on sharing data publicly. For information on unacceptable data access restrictions, please see http://journals.plos.org/plosone/s/data-availability#loc-unacceptable-data-access-restrictions.**

**Authoress’s Response**

There are no legal or ethical restrictions

4. **Review Comments to the Author**
   **Reviewer #1:**

Thanks for reviewer 1 for the good effort to examine our MS.

1. **The literature discussed in the introduction as well as in discussion section needs to be improved for the BPA and BPA related chemicals e.g.BPS etc. There have been some papers recently which can be taken into consideration. The DOI of the articles are https://doi.org/10.1155/2019/9292316 and** [**https://doi.org/10.1007/s11356-022-21672-2**](https://doi.org/10.1007/s11356-022-21672-2)

**Authoress’s Response**

The **introduction was modified.**

2. **Please provide a justification or specific reason to study only the Spleen for the current study. Why they ignored liver as a matter-of-fact toxicant metabolism mostly occurs in liver. Please provide specific reason for eliminating or not considering the liver. Or if you can include liver in the study, it will for sure improve the quality of the manuscript.**

**Authoress’s Response**

BPA exposure has detrimental effects on the vital organs in humans and animals. Since the spleen includes vascular and lymphoid components and is a major site for activating primary immune responses ([Suttie, 2006](https://www.ncbi.nlm.nih.gov/pmc/articles/PMC8956241/#ref36); [Dong et al., 2013](https://www.ncbi.nlm.nih.gov/pmc/articles/PMC8956241/#ref12)), there is increased interest in ascertaining molecular mechanisms and mode of actions underlying spleen pathology induced by BPA.

3. **Please clarify "DNA Genomic Analysis". Genomic analysis means totally different what has been presented in this article. The result shows the integrity of DNA but not the genomics. Therefore, this section needs to be revised with specific language for the method used. Please also include what was the quantity of the DNA used for this test. How it was ensured the all the sample should carry equal amount of DNA?**

**Authoress’s Response**

The DNA genomic analysis has been replaced with analysis of DNA integrity

4. **The article has been corrected for language by the professional services, however, the figures that are included in the manuscript needs revision and attention. The graphs are stretched without keep the constant ratio between width and height. The histopathological figures are stained good however, the photography needs improvement. The scale bars are not labeled to indicated what measurement the bar refers too, though, the captions are well elaborated. The figures are labeled with a group "SHAM" however no such group is elaborated in the text section. Please make the relevant corrections in the figures or the text so as the graphs and the text is in agreement when it comes to talk about different experimental groups.**

**Authoress’s Response**

The text has been adjusted.

5. **In order to improve the quality of the manuscript and its pictorial reflection, the graphs and figures need revision.**

**Authoress’s Response**

The graphs and figures have been revised.

**Reviewer #2:**

Thanks for reviewer 2 for the good effort to examine our MS.

1. **The rationale for the experimental design is not clear in both abstract and introduction. It should be very well clarified.**

**Authoress’s Response**

The rationale for the experimental design has been revised.

2. **This manuscript needs language and grammar editing.**

**Authoress’s Response**

The manuscripts language has been edited.

3. **It is recommended to specify the species of study in the title**

**Authoress’s Response**

The title has been adjusted.

4. **Line 34-35: Abstract can't stand alone, total number of animals used, group size, group names needed to be more illustrated.**

**Authoress’s Response**

The title has been adjusted.

5. **Aim of the study is not clarified, authors don’t illustrate that BPA induced spleen injury and trial to alleviate such detrimental effects. Authors specify that BPA alters Vit D thus, authors should illustrate why study spleen injury no other organs targeted by BPA. The main problem or study aim is not clarified.**

**Authoress’s Response**

BPA exposure has detrimental effects on the vital organs in humans and animals. Since the spleen includes vascular and lymphoid components and is a major site for activating primary immune responses ([Suttie, 2006](https://www.ncbi.nlm.nih.gov/pmc/articles/PMC8956241/#ref36); [Dong et al., 2013](https://www.ncbi.nlm.nih.gov/pmc/articles/PMC8956241/#ref12)), there is increased interest in ascertaining molecular mechanisms and mode of actions underlying spleen pathology induced by BPA.

6. **Line 35: Why authors mentioned 1α, 25-(OH) 2D3 then mentioned Vit D, did they mean its Vit D metabolite or parent form. Write full term at first mention in the text then use abbreviations.**

**Authoress’s Response**

The word 1α, 25-(OH) 2D3 was replaced for consistency.

7. **Line 39: authors didn't mention the age of mice at the start of the experiment, please illustrate what do you meant by this age**

**Authoress’s Response**

The age of animals was included

8. **The BPA could enter organisms through the digestive tract, respiratory tract and skin absorption, of which the digestive tract is the largest source of absorption. Why BPA was administered intraperitoneally in current trial. Please illustrate why this route of exposure**

**Authoress’s Response**

In the current study we sought to explore efficacy of VitD against BPA-induced spleen injury in a mouse model as well as characterized the underlying molecular mechanism(s).

9. **Abstract, unfortunately can't stand alone, several points should be clarified for more illustration such as total number of experimental animals and groups. The measurements that done. Significance of the obtained results like those of lipid peroxidation, DNA fragmentation.**

**Authoress’s Response**

The abstract was revised.

10. **Conclusion in Abstract should be improved.**

**Authoress’s Response**

The abstract was revised.

**Introduction:**

11. Need more reviewing data about the toxic effects of the BPA regarding the spleen injury induced by BPA and not cover the study problem completely. Introduction should include some data refer to BPA and why authors particularly chose this model (mice in this age 3.5 weeks). I am confusing regarding the rationale and the aim of the study.

**Authoress’s Response**

The rationale and the aim of the study was revised.

12. **Line 53-56, 69-71: these paragraphs is away from the aim, design of your current study. Did you investigate any developmental anomalies? Authors should focus on the developmental period and age of mice used in the current trial.**

**Authoress’s Response**

The text has been revised.

13. **Authors are talking about oxidative stress and ROS without owing them to BPA exposure in previous studies**

**Authoress’s Response**

The text has been revised.

**Material and methods section:**

14. **Line 104: authors should mention total number of animals used, sex, and average body weights.**

**Authoress’s Response**

Total number of animals used, sex, and average body weights were included.

15. **Line 115-116: name of experimental groups should be clarified, the full name of the vehicle should be specified, Vit D mentioned into 2 different names in the abstract mentioned as 1α, 25-(OH) 2D3**

**Authoress’s Response**

The word 1α, 25-(OH) 2D3 was replaced for consistency.

16. **Authors mentioned in line 104 that mice were at age of 3.5 weeks then at line 122 at 10.5 weeks and the duration of exposure was 6 weeks which is different to the mentioned ages.**

**Authoress’s Response**

The text was modified.

17. **It is very important to mention reference for oral LD50 that used in current study or your preliminary study citation.**

**Authoress’s Response**

The dose of BPA (50 μg/kg) was chosen based on previous studies ([Sadowski *et al.*, 2014](https://www.ncbi.nlm.nih.gov/pmc/articles/PMC8956241/#ref31)) and Al-Griw et al., 2022 (doi: [10.5455/OVJ.2022.v12.i1.4](https://doi.org/10.5455%2FOVJ.2022.v12.i1.4)).

18. **Line 123-124: For sample collection and preservation, please illustrate types of samples collected and the preservation method or type of preservatives used and for what measurements the samples are collected.**

**Authoress’s Response**

The sample types and preservation method were included (see Peripheral blood and tissue harvesting in the methods section).

19. **For oxidative stress related indices why lipid peroxidation only measured what about other antioxidant enzymes, glutathione, protein carbonyl, total antioxidant capacity, and specific marker of oxidative DNA damage (8-hydroxy-2-deoxyguanosine)**

**Authoress’s Response**

The Lipid peroxidation was only used as biomarkers for oxidative stress injury.

20. **Line 145-147: authors mentioned that blood collected, left to clot to obtain serum. Does leukocytic count done in serum? Or in whole blood also, if whole blood collected please mention type of anticoagulant used.**

**Authoress’s Response**

The EDTA containing tube was used to collect the blood.

21. **For lipid peroxidation MDA measures in spleen homogenate? so, why serum is collected.**

**Authoress’s Response**

The text was modified.

**For results:**

22. **Figure 1 is not cited in the text**

**Authoress’s Response**

The figure 1 was included in the text.

23. **The mean values of the obtained results of all measurements should be mentioned in the result section.**

**Authoress’s Response**

The all measurements have been included in the result section.

**The obtained results, all the measured parameters should be presented as the difference between treatment groups then the difference between sexes.
Authoress’s Response**

The measured parameters have been included.

**Discussion section:**

24. **Firstly, authors should discuss the observed changes in behavioral observations like increased motor activity**

**Authoress’s Response**

The section related to observed alterations in motor activity has been revised.

25. **Line 296-297 authors states that BPA decreased body and spleen weight which in conversely matched with those obtained results in line 227-229
    Authoress’s Response**

The text was modified.

26. **Not all the significantly obtained results are fully discussed**

**Authoress’s Response**

The significant findings have been discussed in the discussion section.

27. **After carefully reinvestigating their preliminary observations, the author could improve the quality of discussion better by integrating the results with the current literature for each discussed parameter.**

**Authoress’s Response**

The discussion section has been revised and improved.

28. **Conclusion is too long**

**Authoress’s Response**

The conclusion has been revised and reduced.

**Reviewer #3:**

Thanks for reviewer 3 for the good effort to examine our MS.

1. **In introduction the author mentioned about the negative effects of vitamin D, briefly explain the negative effects.**

**Authoress’s Response**

There is no mention to the VitD negative effect. We say “Recently, a negative correlation between VitD levels and BPA exposure was reported”

2. **In material and methods author didn’t mention the detail information about the diet given to mice.**

**Authoress’s Response**

All animals used in the current study received a standard diet and drinking water ad libitum.

3. **In statical analysis the author performed two-way ANOVA analysis, a suggestion of performing one-way ANOVA comparison within the groups**

**Authoress’s Response**

To determine statistically significant changes between the treatment groups and control within male and female rats, a two-way ANOVA with Dunnett’s posttest was used.

4. **Why the author chooses 50ug of BPA dose.**

**Authoress’s Response**

It is the daily safety dose. The dose of BPA (50 μg/kg) was chosen based on previous studies ([Sadowski *et al.*, 2014](https://www.ncbi.nlm.nih.gov/pmc/articles/PMC8956241/#ref31)) and Al-Griw et al., 2022 (doi: [10.5455/OVJ.2022.v12.i1.4](https://doi.org/10.5455%2FOVJ.2022.v12.i1.4)).

5. **Mostly the BPA and Vitamin doses are administered orally, why the author choose to inject intraperitoneally.**

**Authoress’s Response**

We administered the BPA and VitD intraperitoneally rather than orally to animals for shorter periods and the absorption rate is higher.

6. **The discussion is quiet long, it’s better to concise it.**

**Authoress’s Response**

The discussion was revised and reduced.
