# Supplementary material for: Therapeutic potential of vitamin D against bisphenol A-induced spleen injury in Swiss albino mice
Source: PLoS One. 2023 Mar 9;18(3):e0280719. doi: 10.1371/journal.pone.0280719 (PMC9997876; doi:10.1371/journal.pone.0280719)
Supplement: Attachment — Submitted filename: Shehreen Amjad comments.docx [file pone.0280719.s005.docx]

Shehreen Amjad

The authors of the manuscript entitled “ **Therapeutic potential of vitamin D against bisphenol A induced spleen injury”** is well written and easy to understand. The manuscript could be considered for publication provided the authors can address major issues listed below.

- The author mentioned about the standard diet. Kindly include the components or company name from where they are getting the feed.
- The biological mechanism of males and females are quite different, why author choose to compare between males and females
- To support the date, it’s better to check the activity of phospho tyrosine and overall PKA specific kinase activity in spleen through western blot to identify certain mechanism involved.
