# Supplementary material for: Therapeutic potential of vitamin D against bisphenol A-induced spleen injury in Swiss albino mice
Source: PLoS One. 2023 Mar 9;18(3):e0280719. doi: 10.1371/journal.pone.0280719 (PMC9997876; doi:10.1371/journal.pone.0280719)
Supplement: Attachment — Submitted filename: Reponse to reviewers _ 20.11.2022.docx [file pone.0280719.s006.docx]

20 November 2022

**Manuscript ID:** PONE-D-22-20374R1

**Manuscript title:** “Therapeutic potential of vitamin D against bisphenol A induced spleen injury”

**Response to reviewers**

**Comment-1**

- The author mentioned about the standard diet. Kindly include the components or company name from where they are getting the feed.

**Response-1**

The diet was purchased from Research Diet Inc.

**Comment-2**

- The biological mechanism of males and females are quite different, why author choose to compare between males and females. To support the date, it’s better to check the activity of phospho tyrosine and overall PKA specific kinase activity in spleen through western blot to identify certain mechanism involved.

**Response-2**

Of course, the biological mechanisms of mouse males and females are different and it was reported that they repose to the bisphenol A (BPA) differently, therefore, we sought in this work to reinvestigate the effect of BPA on both males and females as well as to explore the role of VitD in BPA-induced oxidative splenic injury.
